# Laparoscopic repair of internal hernia in one anastomosis gastric bypass (OAGB): a case report

**DOI:** 10.1093/jscr/rjad670

**Published:** 2023-12-14

**Authors:** Ahmad Essam Al-Mulla, Mohamed Elgazzar, Omar Shalaby

**Affiliations:** Department of Surgery, Farwaniya Hospital, Ministry of Health Kuwait (MOH), Sabah Al-Nasser, Block 6, PO Box 13373, Farwaniya 81004, Kuwait; Department of Surgery, Farwaniya Hospital, Ministry of Health Kuwait (MOH), Sabah Al-Nasser, Block 6, PO Box 13373, Farwaniya 81004, Kuwait; Department of Surgery, Farwaniya Hospital, Ministry of Health Kuwait (MOH), Sabah Al-Nasser, Block 6, PO Box 13373, Farwaniya 81004, Kuwait

**Keywords:** OAGB, internal hernia, bariatric surgery, obesity, small bowel obstruction, mini-gastric bypass surgery

## Abstract

One anastomosis gastric bypass (OAGB), considered an alternative to Roux-en-Y gastric bypass, is becoming an increasingly common procedure. It shows excellent results in terms of weight reduction and remission of metabolic disease. Among the advantages of OAGB is the lack of internal hernia due to the absence of jejuno-jejunal anastomosis. However, internal herniation in OAGB is not impossible, and multiple cases have been mentioned in the literature. We describe a laparoscopic revisional surgery of internal hernia in a patient with a 2-month history of OAGB.

## Introduction

Internal hernia (IH) is a common complication of bariatric surgery. It occurs mainly in Roux-en-Y gastric bypass (RYGB) patients (0.1–12%) [1], either at the defect created by the jejuno-jejenal anastomosis or in Petersen’s space, between the anti-colic Roux-limb and mesentery of the transverse colon [2]. This iatrogenic defect can be dangerous, leading to bowel obstruction and possible ischaemia. One anastomosis gastric bypass (OAGB) is a relatively new procedure, but it is becoming increasingly popular and frequently performed as sleeve gastrectomy and RYGB [3]. OAGB has many advantages in treating obesity, with less incidence of IH. Still, a few cases of IH in OAGB have been reported. Here, we describe the presentation and management of a 50-year-old female who presented with bowel obstruction 2 months after OAGB due to an IH.

## Case report

**Figure 1:**
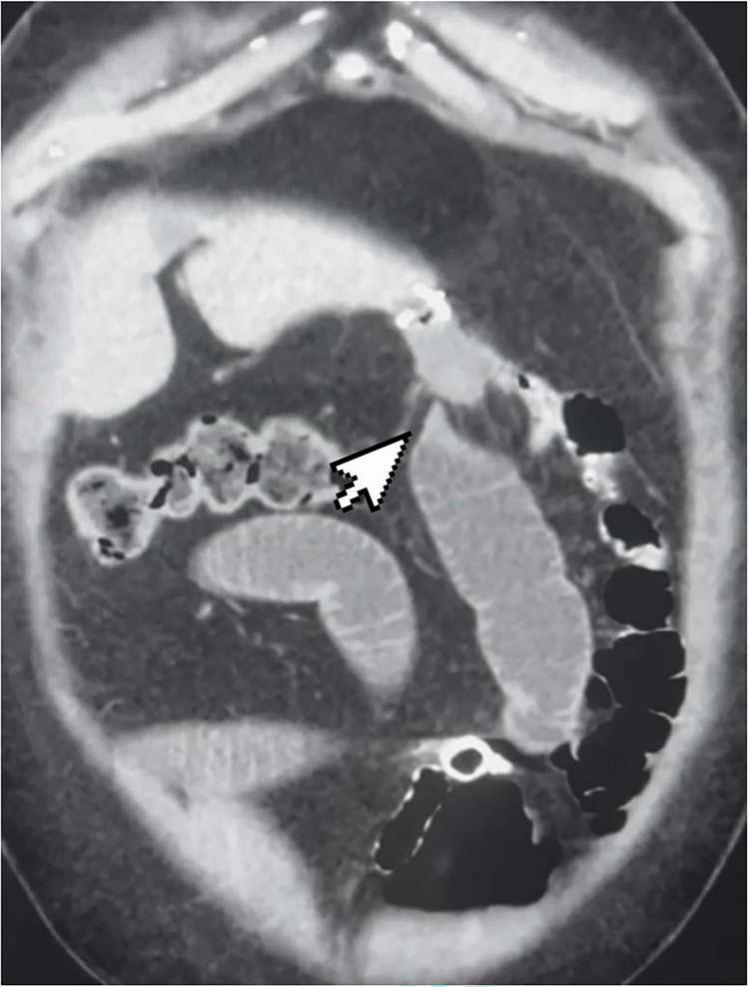
CT scan showing high-grade small bowel obstruction and twisting at the gastro-jejunal anastomosis.

A 50-year-old woman presented to the emergency department complaining of a 3-day history of colicky abdominal pain associated with repeated vomiting and constipation. The patient had a history of OAGB and laparoscopic cholecystectomy 2 months before presentation. During the examination, she was conscious and had normal vital signs. An abdominal examination revealed mild distention and tenderness in the epigastric region. An x-ray of the abdomen showed minimal air-fluid levels. Laboratory tests were unremarkable, except for lipase 135 and C-reactive protein 54. The patient consented to an abdominal computer tomography (CT) scan with intravenous and oral contrast. The CT scan revealed a high-grade obstruction at the jejunum afferent loop with a maximum diameter of 4 cm. No fluid collection, mural thickening, oedema, or fat stranding were detected (Fig. 1). A nasogastric tube was inserted, but no relief or progression was seen in the patient’s situation. The possibility of surgery was raised to the patient, and after mutual agreement, anaesthesiology assessed her for diagnostic laparoscopy.

### Intra-operative

**Figure 2:**
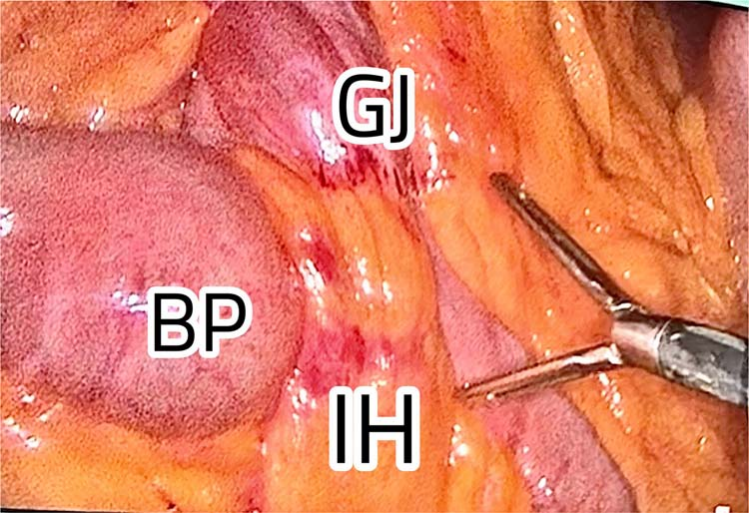
IH at the gastro-jejunal anastomosis.

**Figure 3:**
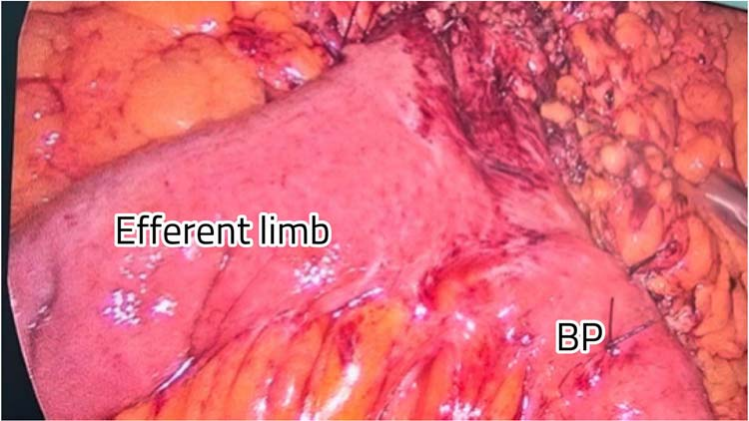
Restoration of the anatomy, fixation, and closure of Petersen’s space.

The patient was positioned with split legs. Five laparoscopic ports were used, and insufflation was obtained. Internal herniation was found at the site of the gastro-jejunal anastomosis, showing an apparent mesenteric twist due to adhesions found at Petersen’s space (Fig. 2). Adhesiolysis was carried out by extending the space, allowing the bowel limbs to un-twist, and placing them in the appropriate direction. The Petersen’s space was closed, and both limbs were fixed to adjacent structures to prevent future herniation. A methylene blue test was performed to check the integrity of the anastomosis. All the bowel loops were examined for adhesions and proper configuration (Fig. 3).

### Postoperative

The patient had an uneventful recovery. The nasogastric tube was removed the next day, and she started a clear fluid diet. She was encouraged to mobilize to prevent future ileus and deep venous thrombosis. On the third postoperative day, the patient complained of severe colicky pain; this was managed conservatively with analgesia and anti-emetic medications. Gastrografin imaging was performed, revealing patent anastomosis with no signs of obstruction, or narrowing. The patient was discharged on the fourth day with instructions to follow a prolonged fluid diet and take proton pump inhibitors. The patient’s first visit to the surgical outpatient department was similarly uneventful, with clean wounds and good weight loss progress.

## Discussion

OAGB is becoming a popular procedure due to its excellent weight reduction results, shorter learning curve, and reduced operating time in theatre compared with RYGB. It is accepted worldwide as an alternative to RYGB. Another significant advantage of OAGB is its lower incidence of IH. This is due to a longer gastric pouch than RYGB, a lack of jejunal-jejunal anastomosis and, therefore, a larger Peterson’s space [4, 6]. A retrospective study of 3368 patients undergoing OAGB found only a 2.8% incidence of IH [5]. Nevertheless, the creation of a mesenteric defect in OAGB procedures still raises the possibility of an IH.

The lack of IH in OAGB studies could be due to the shortness of the follow-up period or the predominance of self-limiting or resolving abdominal symptoms, which are difficult to recognize [8]. Also, the clinical presentation of IH in OAGB can be ambiguous and chronic, with a wide range of possible diagnoses, such as biliary colic, marginal ulcer, anastomotic leakage, pancreatitis, or small bowel adhesions.

Another area for improvement is that computer tomography (CT scan) sensitivity and specificity are only 79 and 59%, respectively [5]. This leads us to question whether the closure of Petersen’s space in OAGB should be necessary. Insufficient evidence supports it, so the closure of the space is not one of the procedural steps [7]. Furthermore, closure of the defect may lead to additional complications, such as mesenteric haematoma, bleeding, kinking, rotation of the anastomosis, and adhesion formation [9]. Nevertheless, the critical lesson is to recommend prompt surgery in cases of high suspicion and chronic abdominal pain to avoid future complications. Up to 20% of IH cases can have normal findings on CT scans and small bowel series [10].

## Conclusion

IH in OAGB is a rare presentation resulting from a significant defect created by gastro-jejunal anastomosis. Due to a lack of evidence, the closure of the space remains an area of discussion and depends solely on the surgeon’s practice. IH should be considered in cases of ambiguous and chronic abdominal pain in OAGB, and prompt surgery should be recommended to avoid adverse complications.
